# Nonalcoholic fatty liver disease is associated with an increased risk of heart block in hospitalized patients with type 2 diabetes mellitus

**DOI:** 10.1371/journal.pone.0185459

**Published:** 2017-10-05

**Authors:** Alessandro Mantovani, Riccardo Rigolon, Isabella Pichiri, Stefano Bonapace, Giovanni Morani, Giacomo Zoppini, Enzo Bonora, Giovanni Targher

**Affiliations:** 1 Section of Endocrinology, Diabetes and Metabolism, Department of Medicine, University and Azienda Ospedaliera Universitaria Integrata of Verona, Verona, Italy; 2 Division of Cardiology, ‘‘Sacro Cuore” Hospital, Negrar, Verona, Italy; 3 Section of Cardiology, Department of Medicine, University and Azienda Ospedaliera Universitaria Integrata of Verona, Verona, Italy; Yonsei University College of Medicine, REPUBLIC OF KOREA

## Abstract

Recent studies suggested that nonalcoholic fatty liver disease (NAFLD) is associated with an increased risk of cardiac tachyarrhythmias (mainly atrial fibrillation) in patients with and without type 2 diabetes mellitus. The aim of this study was to examine whether an association also exists between NAFLD and heart block. We have retrospectively evaluated a hospital-based cohort of 751 patients with type 2 diabetes discharged from our Division of Diabetes and Endocrinology during years 2007–2014. Standard electrocardiograms were performed on all patients. Diagnosis of NAFLD was based on ultrasonography, whereas the severity of advanced hepatic fibrosis was based on the fibrosis (FIB)-4 score and other non-invasive fibrosis markers. Overall, 524 (69.8%) patients had NAFLD and 202 (26.9%) had heart block (defined as at least one block among first-degree atrio-ventricular block, second-degree block, third-degree block, left bundle branch block, right bundle branch block, left anterior hemi-block or left posterior hemi-block) on electrocardiograms. Patients with NAFLD had a remarkably higher prevalence of any persistent heart block than those without NAFLD (31.3% *vs*. 16.7%, *p*<0.001); this prevalence was particularly increased among those with higher FIB-4 score. NAFLD was associated with a threefold increased risk of prevalent heart block (adjusted-odds ratio 3.04, 95% CI 1.81–5.10), independently of age, sex, hypertension, prior ischemic heart disease, hemoglobin A1c, microvascular complication status, use of medications and other potentially confounding factors. In conclusion, this is the largest cross-sectional study to show that NAFLD and its severity are independently associated with an increased risk of prevalent heart block in hospitalized patients with type 2 diabetes.

## Introduction

Nonalcoholic fatty liver disease (NAFLD) has emerged as an imperative public health problem worldwide. NAFLD represents the most common chronic liver disease in high-income countries, and is estimated to affect at least 25–30% of the general adult population and up to 75–80% of patients with type 2 diabetes mellitus (T2DM) [1,2]. Patients with T2DM are also more likely to develop the more severe histological forms of NAFLD (*i*.*e*., steatohepatitis [NASH], advanced fibrosis and cirrhosis) [1,2].

Over the past decade, increasing evidence has suggested that NAFLD is a multi-system disease that affects not only the liver but also the cardiovascular system, leading to functional and structural changes both in the heart and in the blood vessels. Ultimately, these changes may be responsible for the increased cardiac morbidity and mortality associated with NAFLD [3–5]. In line with this conclusion, given that cardiovascular disease (CVD) complications frequently dictate the outcome(s) of patients with NAFLD, the 2016 European clinical practice guidelines for the management of NAFLD have strongly recommended CVD risk assessment in all patients with NAFLD [6].

Accumulating evidence now also substantiates the existence of a link between NAFLD and cardiac arrhythmias [7]. In particular, several epidemiological studies have suggested that NAFLD is strongly associated with an increased risk of permanent atrial fibrillation, heart rate-corrected QT interval prolongation and ventricular tachyarrhythmias both in patients with and without T2DM [8–13]. To date, only one retrospective case-control study has ascertained the association between NAFLD and heart block, demonstrating that NAFLD is closely associated with the presence of cardiac conduction defects in a hospital-based sample of 700 adult patients (about 30% of them had known T2DM) admitted to the Orange Park Medical Center in Florida from 2009 to 2015 [14].

Currently, it is uncertain whether there is an association of NAFLD and its severity with cardiac conduction defects also in people with T2DM. We believe that this topic merits further in-depth investigation as it might contribute to explain the increased risk of cardiac morbidity and mortality observed in patients with NAFLD. Indeed, while it is well known that high-degree atrio-ventricular (AV) blocks are powerful predictors of CVD mortality in affected patients [15,16], there is now emerging evidence suggesting that prolonged PR interval, first-degree AV block and RBBB are also associated with poor cardiac prognosis [17–19]. For example, in a meta-analysis of 14 large observational studies (involving a total of nearly 400,000 individuals), Kwok *et al*. reported a significant association between PR interval prolongation or first-degree AV block and increased risks of atrial fibrillation, heart failure and mortality [19].

Thus, the main purpose of this study was to examine whether ultrasound-diagnosed NAFLD and its severity (using non-invasive markers of advanced NAFLD fibrosis)[20] are associated with an increased prevalence of heart block in a large sample of hospitalized patients with T2DM.

## Materials and methods

### Patients

For this registry study, we have retrospectively identified all patients with established T2DM, who were discharged from our Division of Diabetes and Endocrinology at the Verona University hospital during the years 2007–2014. Where a patient had had multiple discharges in 2007–2014 the first discharge with complete data was considered for the statistical analysis. Most of these patients were admitted to the hospital for the following main clinical reasons: chronic decompensated diabetes, diabetic foot ulcers or infections.

**Fig 1:**
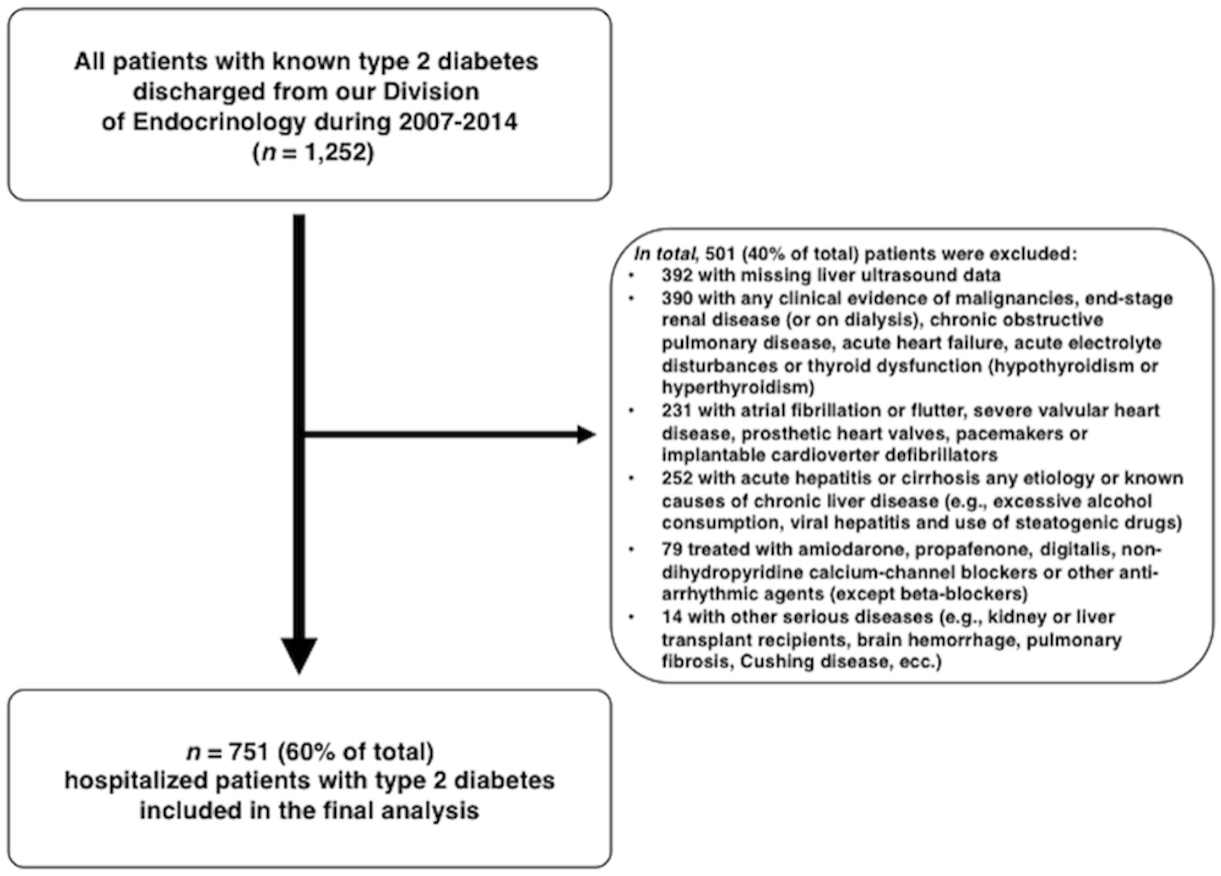
The flowchart shows the details of the study design.

A total of 1,252 hospitalized patients were initially identified in our electronic database. We subsequently excluded 501 patients with excessive alcohol consumption (defined as >20 g/day of alcohol for women and >30 g/day for men, respectively) [6], viral hepatitis, drug-induced liver disease, cirrhosis of any etiology or other important comorbid conditions (including pre-existing atrial fibrillation or flutter, pacemakers or implantable cardioverter defibrillators, severe valvular heart disease, malignancies, end-stage renal disease, chronic obstructive pulmonary disease, acute electrolyte disturbances or thyroid dysfunction) as well as patients taking any anti-arrhythmic drugs (except beta-blockers) or with missing liver ultrasound data. More details about the study design and the reasons of exclusion from the study are reported in Fig 1.

As a result of this selection, 751 (60% of total) patients met the inclusion criteria of the study and were included in the final analysis.

The local ethics committee/IRB of the Verona University Hospital approved the study protocol. The ethics committee exempted our research from the informed consent requirement because we only accessed retrospectively a de-identified database for the purpose of data analysis.

### Clinical and laboratory variables

Patients were considered to have hypertension if their blood pressure was ≥140/90 mmHg or if they were taking any anti-hypertensive agents. Detailed information on comorbid conditions, use of medications and daily alcohol consumption was acquired in all patients by interviews during medical visits. In particular, alcohol consumption was assessed on the basis of the self-reported number of drinks consumed per day. The following amounts of alcoholic beverages were considered 1 drink: 330 ml beer (containing ∼5% of alcohol), 150 ml wine (containing ∼12% of alcohol), and 40 ml strong alcohol (containing ∼50% of alcohol). By study design, most patients included in this analysis were alcohol abstainers (nearly 80% of total), whereas the remaining individuals drank less than 20 g/day of alcohol if women or less than 30 g/day of alcohol if men.

Complete blood count, serum liver enzymes (*i*.*e*., alanine aminotransferase [ALT], aspartate aminotransferase [AST] and gamma-glutamyltransferase [GGT]), creatinine, electrolytes, thyroid-stimulating hormone and other biochemical blood measurements were determined using standard laboratory procedures. Hemoglobin A1c (HbA1c) was measured by a high-performance liquid chromatography analyzer on Tosoh-G7 automated analyzer (Tosoh Bioscience Inc., San Francisco, CA; USA); the upper limit of normal for our laboratory was 5.6%. Glomerular filtration rate (eGFR) was estimated by using the Chronic Kidney Disease Epidemiology Collaboration (CKD-EPI) equation [21,22]. We also calculated the APRI index (AST to Platelet Ratio Index) by using the formula: (AST/upper limit of normal x 100)/platelet count), and the fibrosis (FIB)-4 score by using the formula: age × AST (IU/l)/platelet count (×10^9^/l) × √ALT (IU/l), which are non-invasive markers of advanced liver fibrosis [20]. For the FIB-4 score, we used the new cutoffs proposed for NAFLD patients aged ≥65 years [23]. Both of these non-invasive fibrosis scores were calculated only in the subgroup of patients with NAFLD (*n* = 348), who had available measurements of platelet count and serum AST levels. We did not calculate the NAFLD fibrosis score because serum albumin measurements were available only in few patients.

Albuminuria was measured by an immuno-nephelometric method on a morning spot urine sample and expressed as the albumin/creatinine ratio on Beckman-Coulter IMMAGE (Beckman-Coulter Instruments, Fullerton, CA; USA); macroalbuminuria was defined as an urinary albumin/creatinine ratio >300 mg/g.

Ischemic heart disease was defined as a documented history of myocardial infarction, angina, coronary revascularization procedures or typical electrocardiographic abnormalities (according to the Minnesota code) [22]. Pre-existing history of mild-to-moderate heart valve disease was confirmed by reviewing medical records of the hospital, including diagnostic symptoms patterns and echocardiograms (those with severe valvular diseases or prosthetic heart valves were excluded from the study). The pre-existing history of peripheral artery disease was based on medical history and examination (*e*.*g*., intermittent claudication, rest pain, lower-extremity revascularizations), and was confirmed by reviewing medical records of the hospital of patients, including radiologic imaging results. In most patients, the presence of microvascular diabetic complications such as diabetic retinopathy (by fundoscopy after pupillary dilation) and lower-extremity sensory neuropathy (by biothesiometer or 5.07/10-gm monofilament) were also recorded.

### Electrocardiograms and liver ultrasonography

A standard 12-lead electrocardiogram (ECG) was performed in all patients during the hospital stay (and then repeated when necessary). A 24-hour ECG Holter monitoring was not routinely performed.

The diagnosis of heart block was made on the basis of automatic interpretation of standard ECGs that was subsequently validated by an expert cardiologist, who was blinded to patient’s clinical data. In particular, the first-degree AV block was defined as a PR interval duration of 200 ms or more without variation [24]. The diagnosis of second-degree AV block was made according to the progressive prolongation of PR interval, culminating in a non-conducted P wave. Second-degree AV block was subsequently classified in Mobitz type I or Mobitz type II. A third-degree AV block was diagnosed when the P waves were not followed by QRS complexes [24]. Similarly, the presence of complete right bundle branch block (RBBB), left bundle branch block (LBBB), left anterior hemi-block (LAH or left anterior fascicular block) or left posterior hemi-block (LPH or left posterior fascicular block) were also diagnosed by a cardiologist according to standard ECG criteria [24].

Hepatic ultrasonography was performed in all patients by experienced radiologists, who were blinded to patient’s clinical data. Hepatic steatosis was diagnosed based on characteristic ultrasonographic characteristics, including diffuse hyperechogenicity of the liver relative to the kidneys, ultrasonography beam attenuation, and poor visualization of the intrahepatic vessel borders and diaphragm [25]. Ultrasonography has a good diagnostic accuracy to detect the presence of mild and moderate-to-severe hepatic steatosis, demonstrating a sensitivity and specificity, respectively, of approximately 85% and 95% (when liver fat infiltration on histology is at least 20–30%) [25].

### Statistical analysis

Data are expressed as means±SD, medians and inter-quartile ranges (IQR) or proportions. Differences in clinical, biochemical and electrocardiogram characteristics of patients were assessed using the unpaired Student’s *t* test for normally distributed variables and the Mann-Whitney-U test for non-normally distributed variables. The chi-squared test was used to test for between-group differences among the categorical variables. Logistic regression analysis was used to assess the association between NAFLD and risk of any persistent heart block (defined as presence of at least one block among first-degree AV block, second-degree AV block, third-degree AV block, LBBB, RBBB, LAH or LPH) after adjustment for potential confounding factors. Four forced-entry logistic regression models were performed as follows: the first model was unadjusted; the second model was adjusted for age and sex (model 1); the third model was adjusted for age, sex, BMI, diabetes duration, hemoglobin A1c, eGFR-EPI, macroalbuminuria, hypertension (*i*.*e*., blood pressure ≥140/90 mmHg or use of any anti-hypertensive drugs, *including* also beta-blockers), prior ischemic heart disease and mild-to-moderate valvular heart disease (model 2). Lastly, the fourth model was additionally adjusted for peripheral artery disease, diabetic retinopathy, lower-extremity sensory neuropathy and current use of statins or anti-platelet agents (model 3). Covariates included in multivariate logistic regression models were selected as potential confounding factors based on their significance in univariate analyses or based on their biological plausibility. A *p*-value <0.05 was considered statistically significant.

## Results

A total of 751 adult patients with T2DM were included in the study. All patients were Caucasian. They had a mean age of 66 years and a median duration of diabetes of approximately 15 years. NAFLD was identified in 524 (69.8%) patients. There were 242 total heart block events among 202 (26.9%) patients, who had some type of persistent heart block demonstrated on standard ECGs. In particular, 94 patients had a first-degree AV block, 5 had a second-degree AV block (1 of whom had Mobitz type I and 4 had Mobitz type II), 1 had a third-degree AV block, 67 had RBBB, 17 had LBBB, 56 had LAH and 2 patients had LPH. It is important to remember that, by study design (as summarized in Fig 1), no patients were treated with amiodarone, propafenone, digitalis, non-dihydropyridine calcium-channel blockers or other anti-arrhythmic agents, except beta-blockers.

Table 1 shows the clinical, biochemical and electrocardiogram characteristics of participants stratified by presence/absence of any heart block. Patients with heart block were older, more likely to be male, had longer duration of diabetes, higher serum creatinine, lower eGFR values and longer PR, QRS and QTc intervals on ECGs compared to those without heart block. Moreover, they also had lower levels of HbA1c, total and LDL-cholesterol. Additionally, the prevalence of hypertension, ischemic heart disease, mild-to-moderate valvular heart disease, peripheral artery disease, diabetic retinopathy, lower-extremity sensory neuropathy as well as the proportion using dihydropyridine calcium channel blockers (patients taking non-dihydropyridine calcium channel blockers were excluded from the study), diuretics, nitroderivates, statins or anti-platelet agents were greater in patients with heart block than in those without. Notably, patients with heart block also had a significantly higher prevalence of NAFLD compared with their counterparts without heart block. Conversely, obesity, smoking, albuminuria and the percentage of those treated with renin-angiotensin-aldosterone system inhibitors, beta-blockers or glucose-lowering agents (except for a lower use of metformin) did not significantly differ between the two groups.

**Table 1:** Clinical, biochemical and electrocardiographic characteristics of patients with T2DM stratified by heart block status.

Characteristics	Patients without heart block (*n* = 549)	Patients with heart block (*n* = 202)	*p* value
Age (years)	64.0 ± 13	70.0 ± 11	<0.001
Men/Women (n)	283/266	122/80	<0.05
Body weight (kg)	83.0 ± 21	85.0 ± 23	0.30
BMI (kg/m^2^)	30.4 ± 7	30.5 ± 6.5	0.85
Smokers (%), n = 219	48.1	36.3	0.14
Diabetes duration (years)	13 (6–20)	19 (10–28)	<0.001
Systolic blood pressure (mmHg)	140 ± 20	141 ± 21	0.81
Diastolic blood pressure (mmHg)	81 ± 11	79 ± 12	0.11
Fasting glucose (mmol/l)	9.3 (7.0–13.4)	9.2 (6.7–13.6)	0.98
Hemoglobin A1c (%)	9.9 ± 2.6	9.5 ± 2.4	<0.05
Total cholesterol (mmol/l)	4.56 ± 1.3	4.21 ± 1.2	<0.001
LDL-cholesterol (mmol/l)	2.59 ± 1.1	2.29 ± 0.9	<0.001
HDL-cholesterol (mmol/l)	1.08 ± 0.3	1.05 ± 0.3	0.36
Triglycerides (mmol/l)	1.64 (1.23–2.33)	1.56 (1.08–2.23)	0.32
AST (U/l), n = 483	20 (15–28)	21 (15–30)	0.70
ALT (U/l)	22 (16–33)	20 (14–29)	0.15
GGT (U/l)	31 (18–57)	30 (18–54)	0.45
Creatinine (mmol/l)	95.6 ± 44	110.5 ± 53	<0.001
eGFR-EPI (ml/min/1.73 m^2^)	72.1 ± 24	62.5 ± 24	<0.001
Hemoglobin (g/dl)	13.1 ± 1.8	12.7 ± 1.9	0.06
Platelets (x 10^9^/l)	239 ± 73	236 ± 73	0.63
Hypertension (%)	78.6	86.6	<0.01
Obesity, BMI ≥30 kg/m^2^ (%)	43.9	46.0	0.62
Ischemic heart disease (%)	18.7	25.7	<0.05
Mild-moderate valvular heart disease (%)	7.3	14.8	<0.01
Microalbuminuria (%)	31.0	35.0	0.26
Macroalbuminuria (%)	11.5	10.4	0.69
Diabetic retinopathy (%), any degree	40.3	52.6	<0.01
Diabetic sensory neuropathy (%), n = 731	27.5	37.6	<0.01
Peripheral artery disease (%)	48.4	65.6	<0.001
Insulin users (%)	69.5	75.5	0.11
Metformin users (%)	43.4	33.6	<0.05
Sulfonylurea users (%)	20.2	22.9	0.41
Glitazone users (%)	3.1	2.0	0.62
DPP-4 inhibitor users (%)	9.4	6.6	0.30
GLP-1 analogues users (%)	1.4	1.0	0.80
Acarbose users (%)	2.7	1.5	0.43
ACE-inhibitor users (%)	52.1	55.1	0.47
ARB users (%)	21.8	22.4	0.85
Alpha-blocker users (%)	9.1	11.2	0.36
Beta-blocker users (%)	29.9	32.1	0.56
Dihydropyridine CCB users (%)	30.6	39.3	<0.05
Diuretic users (%)	43.4	59.7	<0.001
Anti-platelet drug users (%)	58.2	76.5	<0.001
Nitroderivate drug users (%)	9.7	16.8	<0.01
Statin users (%)	59.0	66.8	<0.05
Fibrate users (%)	4.3	2.5	0.39
NAFLD (%)	68.7	81.2	<0.001
**Electrocardiographic findings**			
Heart rate (bpm)	75 ± 13	74 ± 12	0.54
PR interval (ms)	159 ± 23	198 ± 43	<0.001
QRS duration (ms)	92 ± 17	113 ± 26	<0.001
QTc interval duration (ms)	432 ± 28	447 ± 48	<0.001

Sample size, *n* = 751 except where indicated.

Data are expressed as means±SD, medians and interquartile ranges (IQR) or percentages. Heart block was defined as the presence on a resting 12-lead ECG of at least one heart block among first-degree AV block, second-degree AV block, third-degree AV block, LBBB, RBBB, LAH or LPH.

Differences between the two groups were tested by the chi-squared test for categorical variables, the unpaired Student’s *t*-test for normally distributed continuous variables or the Mann-Whitney test for non-normally distributed continuous variables.

Abbreviations: ALT, alanine aminotransferase; AST, aspartate aminotransferase; ARB, angiotensin receptor blockers; BMI, body mass index; CCB, calcium channel blockers; DPP-4, dipeptidyl peptidase-4; eGFR-EPI, glomerular filtration rate as estimated by the CKD-EPI equation; GGT, gamma-glutamyltransferase; GLP-1, glucagon-like peptide-1.

S1 Table summarizes the clinical and biochemical characteristics of T2DM patients stratified by NAFLD status. Compared with those without NAFLD, patients with NAFLD were older, more likely to be male and had higher values of BMI, blood pressure, HbA1c, total cholesterol, triglycerides, ALT, GGT and hemoglobin, and lower HDL-cholesterol levels. The prevalence of hypertension, smoking history, and the proportion using metformin, alpha-blockers and statins were also greater in patients with NAFLD. Conversely, duration of diabetes, prevalence of prior ischemic heart disease, mild-to-moderate valvular heart disease, microvascular complication status as well as the use of glucose-lowering agents (except metformin), anti-hypertensive drugs (expect alpha-blockers) or anti-platelet agents did not differ significantly between the two groups of patients.

**Fig 2:**
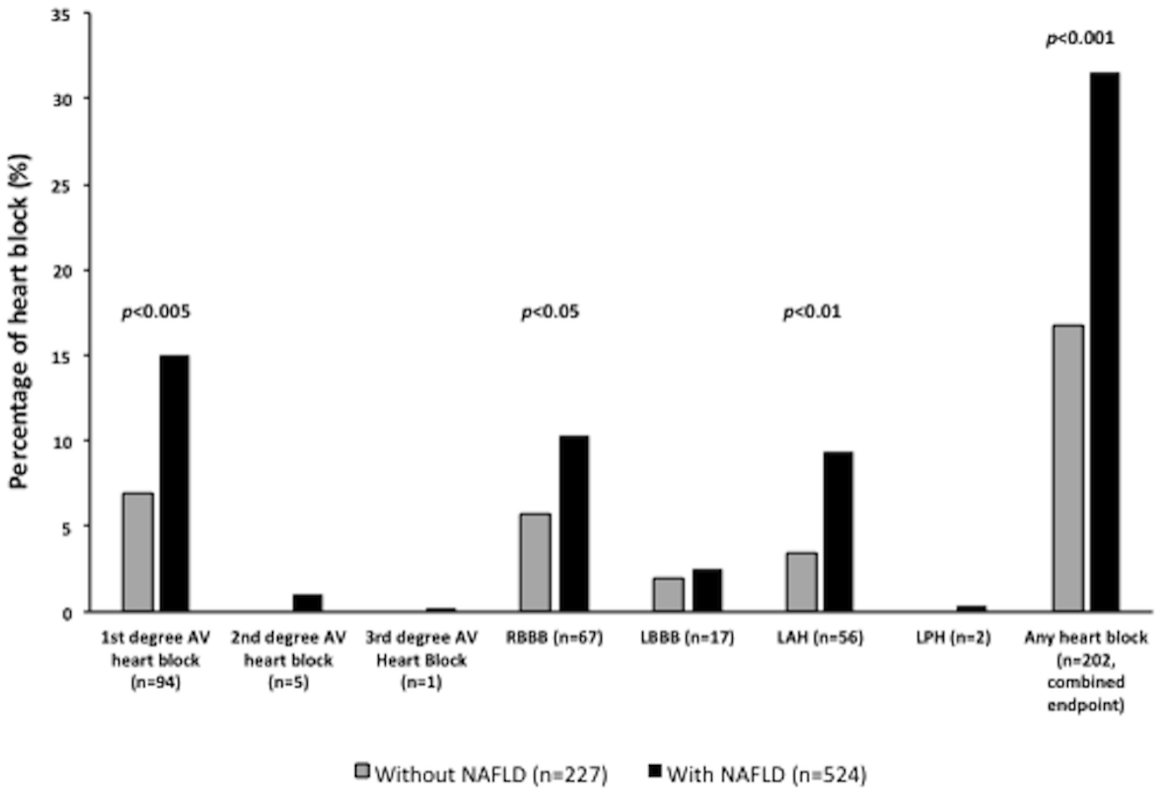
Prevalence of heart block in NAFLD. Prevalence of different types of heart block (singly or in combination) on resting 12-lead electrocardiograms in patients with T2DM stratified by NAFLD status. In the figure have been reported only the *p* values for the inter-group differences that were statistically significant.

Notably, as shown in Fig 2, patients with NAFLD had a remarkably greater prevalence of heart block (combined endpoint) than their counterparts without NAFLD (31.3% *vs*. 16.7%; *p*<0.001). Examining each type of heart block separately, the prevalence of first-degree AV block, LAH and RBBB was higher in patients with NAFLD than in those without. No significant differences were observed in the prevalence of second-degree block, third-degree block, LBBB or LPH between patients with and those without NAFLD.

**Fig 3:**
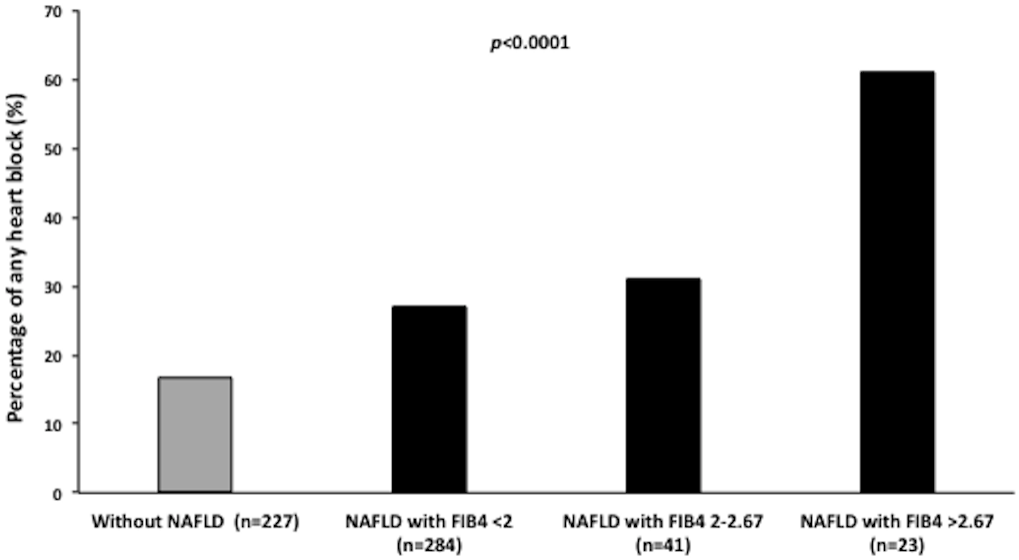
Prevalence of heart block in relation to advanced NAFLD fibrosis. Prevalence of any heart block (combined endpoint) in patients without NAFLD (gray column; *n* = 227) and in patients with NAFLD (black columns; *n* = 348) stratified by fibrosis (FIB)-4 score. *P*-value <0.001 for the unadjusted trend. This trend remained statistically significant even after adjustment for age, sex, BMI, hemoglobin A1c, eGFR, macroalbuminuria, hypertension, prior ischemic heart disease and mild-to-moderate valvular heart disease. Data on FIB-4 score were available only in 348 patients with NAFLD.

As shown in Fig 3, there was a strong, graded relationship between the prevalence of any heart block (combined endpoint) and the severity of NAFLD fibrosis (as estimated by the FIB-4 score). Indeed, patients with NAFLD and high FIB-4 score (FIB-4 >2.67, *i*.*e*., a marker of advanced NAFLD fibrosis) had a substantially greater prevalence of heart block as compared to other subgroups of NAFLD patients with normal or intermediate FIB-4 scores or those without NAFLD (*p*<0.001 for the unadjusted trend). Notably, this trend remained statistically significant even after adjusting for age, sex, BMI, diabetes duration, HbA1c, eGFR, macroalbuminuria, hypertension, prior ischemic heart disease and mild-to-moderate valvular heart disease (*p*<0.01 for the adjusted trend).

Similar results were also observed when we used the APRI index; also in such case, there was an increasing prevalence of any heart block in relation to the severity of NAFLD fibrosis that varied, respectively, from 16.7% in patients without NAFLD to 20% in patients with NAFLD and APRI index <0.5 (*i*.*e*., the lowest score), 22% in patients with NAFLD and APRI index between 0.5 and 1.4 (intermediate score) and 30% in those with NAFLD and APRI index >1.5, *i*.*e*. the highest score (*p*<0.05 for the unadjusted trend); the significance of such relationship was lost after adjustment for potential confounding factors. However, these results should be interpreted with some caution because both FIB-4 and APRI scores have not been sufficiently validated in a non-NAFLD population.

Table 2 shows the effect of the statistical adjustment for multiple potential confounding factors on the association between NAFLD and heart block. In univariate logistic regression analysis (unadjusted model), NAFLD was associated with an approximately 2.3-fold increased risk of prevalent heart block. This association remained significant even after adjustment for age, sex, BMI, diabetes duration, HbA1c, eGFR, macroalbuminuria, hypertension, prior ischemic heart disease and mild-to-moderate valvular heart disease (adjusted models 1 and 2; *n* = 751). Further adjustment for peripheral artery disease, diabetic retinopathy, lower-extremity sensory neuropathy and use of statins or anti-platelet agents did not weaken this association (adjusted model 3; *n* = 731). In this latter model other variables that were independently associated with heart block, together with NAFLD, were older age, male sex, presence of valvular heart disease and lower eGFR values.

**Table 2:** Logistic regression models—Association between NAFLD and the risk of prevalent heart block in patients with T2DM.

Logistic Regression Models	Odds Ratio	95% CI	*p* value
**NAFLD (yes *vs*. no)**			
Unadjusted model (*n* = 751)	2.27	1.53–3.36	<0.001
Adjusted model 1 (*n* = 751)	2.65	1.75–4.01	<0.001
Adjusted model 2 (*n* = 751)	2.82	1.73–4.57	<0.001
Adjusted model 3 (*n* = 731)	3.04	1.81–5.10	<0.001
**Other independent predictors of heart block in regression *model 3***			
Age (years)	1.03	1.01–1.06	<0.001
Sex (men *vs*. women)	1.67	1.10–2.44	<0.01
Mild-moderate heart valve disease (yes *vs*. no)	1.59	1.01–2.98	<0.05
Serum creatinine (mmol/l)	0.98	0.97–0.99	<0.05

Data are expressed as odds ratios ± 95% confidence intervals (CI) as assessed by either univariate (unadjusted) or multivariate logistic regression analyses. The presence of heart block (defined as presence on a resting 12-lead ECG of at least one heart block among first-degree AV block, second-degree AV block, third-degree AV block, LBBB, RBBB, LAH or LPH) was included as the dependent variable in all logistic regression models.Other covariates included in multivariate logistic regression models, together with NAFLD, were as follows: *model 1*: adjusted for age and sex; *model 2*: adjusted for age, sex, body mass index, duration of diabetes, hemoglobin A1c, eGFR-EPI, macroalbuminuria, hypertension status (*i*.*e*., blood pressure ≥140/90 mmHg or use of any anti-hypertensive drugs, including also beta-blockers), prior ischemic heart disease and mild-to-moderate valvular heart disease; *model 3*: adjusted for the same variables included in model 2 *plus* peripheral artery disease, diabetic retinopathy, lower-extremity sensory neuropathy, and current use of statins or anti-platelet agents.

The significant and independent association between NAFLD and risk of any permanent heart block remained essentially unchanged even when we excluded from the analysis the patients who currently smoked (adjusted-OR 2.93; 95% CI 2.8–4.9) or those with previous ischemic heart disease (adjusted-OR 3.51; 95% CI 1.9–6.4) or those treated with dihydropyridine calcium-channel blockers (adjusted-OR 3.03; 95% CI 1.6–5.9) or, finally, patients who were treated with beta-blockers (adjusted-OR 3.94; 95% CI 2.1–7.6). As regards to the use of beta-blockers, it is also important to underline that the proportion using beta-blockers was essentially superimposable between patients with and without NAFLD as well as between those with and without heart block (Table 1 and S1 Table).

We undertook a sensitivity analysis, by repeating the regression models shown in Table 2 where the dependent variable was either the presence of any degree of AV block or the presence of bundle branch block or hemi-block (i.e., BBBB, LBBB, LAH or LPH), instead of the presence of any heart block. Also in such case, the presence of ultrasound-diagnosed NAFLD was associated with a nearly threefold increased risk of prevalent AV block (of any degree) after adjusting for all potential confounding factors listed in the above-mentioned regression model 3 (adjusted-OR 3.07, 95% CI 1.5–6.2; *p*<0.01). Similarly, NAFLD was independently associated with a nearly 2.7-fold increased risk of prevalent bundle branch block or hemi-block (adjusted-OR 2.69, 95% CI 1.5–4.9; *p*<0.01).

## Discussion

The novel findings of our study are as follows: 1) patients with coexistent NAFLD and T2DM had a remarkably higher prevalence of any persistent heart block on resting 12-lead ECGs (principally first-degree AV block, RBBB and LAH) compared with their counterparts without NAFLD; 2) the prevalence of any persistent heart block was particularly increased among those with advanced NAFLD fibrosis (as estimated by the FIB-4 score that performed better than the APRI score in our patients with T2DM); and 3) NAFLD was associated with a threefold higher risk of any persistent heart block even after adjustment for age, sex, diabetes-related variables and a range of other potential confounding factors.

To our knowledge, this is the largest cross-sectional study aimed at assessing whether ultrasound-diagnosed NAFLD and its severity (as estimated by non-invasive fibrosis biomarkers) are associated with the presence of heart block in a large hospital-based sample of patients with T2DM.

Our results confirm and extend the findings recently published by Mangi *et al*. in a case-control retrospective study that included 408 hospitalized adult patients with NAFLD and 292 patients without NAFLD (only 30% of whom had known T2DM), who were admitted to the hospital (at the Orange Park Medical Center in Florida) over a period of 6 years [14]. In such study, NAFLD was identified using the International Classification of Diseases, Ninth Revision (ICD-9) codes; and 140 (20%) patients had some type of persistent heart block demonstrated on standard ECGs (most of whom had RBBB or a first-degree AV block). Notably, the authors found that NAFLD was closely associated with the presence of any persistent heart block on standard ECGs (adjusted-OR 2.38; 95% CI 1.5–3.7), independently of numerous CVD risk factors (including diabetes status, which was also found to be independently associated with heart block) [14].

Overall, therefore, our findings confirm on a large sample of hospitalized patients with established T2DM the results recently published by Mangi *et al*. [14]. In addition, we showed for the first time that the prevalence of cardiac conduction defects was particularly increased in patients with advanced NAFLD fibrosis (as estimated by the FIB4 score or the APRI index). These latter results are consistent with the notion that it is advanced NAFLD (*i*.*e*., fibrosing liver disease) which is more strongly associated with the risk of developing extra-hepatic (notably including cardiovascular) complications among patients with NAFLD [2,5,7,26].

At present, convincing evidence substantiates the existence of a link of NAFLD with ischemic heart disease and the presence of both cardiomyopathy (*i*.*e*., left ventricular diastolic dysfunction and hypertrophy) and heart valve calcification (*i*.*e*., aortic-valve sclerosis and mitral annulus calcification, which are also established risk factors for cardiac conduction defects) [3–5,7,27–30]. More recently, some epidemiological studies also documented the existence of a significant and independent association of NAFLD with permanent/persistent atrial fibrillation [8,9]; electrocardiographic QTc interval prolongation [11,12]; cardiac autonomic dysfunction [31]; and presence of ventricular tachyarrhythmias on 24-hour ECG Holter monitoring [13].

Collectively, we believe that these findings may have important clinical implications. Firstly, our study expands previously published observations regarding a possible arrhythmogenic role of NAFLD. Secondly, the existence of an association between NAFLD and cardiac conduction defects may contribute to further explain the increased risk of fatal and nonfatal CVD events observed in patients with NAFLD. Thirdly, our data further reinforce the clinical importance of implementing screening of the cardiovascular system in all patients with NAFLD [4,6].

The underlying pathophysiological mechanisms responsible for the observed association between NAFLD and heart block are not completely understood. It remains uncertain if NAFLD is a simple risk marker for the development of CVD and other cardiac and arrhythmic complications or if NAFLD is a direct contributor to the development and progression of these cardiac and arrhythmic complications. An obvious explanation for these results is that the association we observed between NAFLD and heart block simply reflects the coexistence of multiple CVD risk factors and comorbidities. However, it is important to underline that in our study the significant association between NAFLD and heart block persisted even after adjustment for multiple established risk factors and potential confounders (including also previous ischemic heart disease and presence of lower-extremity sensory neuropathy, which is thought to be a reliable marker of cardiovascular autonomic neuropathy [32]). This finding, therefore, suggests the possibility that NAFLD *per se* might play a role in the development and persistence of heart block. As previously mentioned, clear evidence indicates that NAFLD is not only associated with an increased risk of CVD morbidity and mortality, but is also associated with the presence of functional and structural cardiomyopathy that may lead to the development of electrophysiological disorders of the heart [7]. Accumulating evidence from studies performed in both nondiabetic and diabetic patients indicates that the presence of NAFLD (especially NASH with varying amounts of hepatic fibrosis) exacerbates hepatic/peripheral insulin resistance and causes the systemic release of multiple proinflammatory factors, vasoactive mediators and thrombogenic molecules that are important in the development of cardiovascular disease and other functional, structural and arrhythmic complications of the heart [3,4,7]. It is plausible to assume that all these NAFLD-related myocardial changes may also promote progressive fibrosis of the His-Purkinje system and deep derangements in its distribution and transmission velocities, producing a delay or even a stop of the impulse conduction across the AV node, His bundle and bundle branches. Although these findings require further testing and confirmation in larger studies, however, we believe that the pathophysiological pathways through which NAFLD contributes to systemic chronic inflammation, hypercoagulation and insulin resistance might represent potential therapeutic targets for the prevention and treatment of myocardial remodeling and the electrophysiological abnormalities of the myocardium in patients with NAFLD [7].

There are limitations and strengths to our study that should be considered. Firstly, this study is limited by its single-center, retrospective design, which limits our ability to draw any firm conclusion about the temporality and causality of the observed associations. Secondly, although we excluded most patients with serious comorbidities, however, we cannot definitely exclude a possible selection bias of including a hospital-based sample of patients with T2DM; so these results might not directly be generalizable to other diabetic populations. Thirdly, although our logistic regression models were extensive, we cannot exclude the possibility of residual confounding by some unmeasured factors that might partly explain the observed associations. Fourthly, the diagnosis of NAFLD was based on ultrasonography and exclusion of secondary causes of chronic liver disease but was not confirmed by liver biopsy, which is considered as the ‘reference standard’ for diagnosing and staging NAFLD. However, ultrasonography is the recommended first-line imaging modality for detecting NAFLD in clinical practice [6], and it enables a reliable and accurate detection of mild-to-moderate hepatic steatosis compared with liver histology [25]. Finally, the use of non-invasive markers of advanced NAFLD fibrosis, such as the FIB-4 and APRI scores, has not been adequately validated in people with T2DM or in a general population. Future studies in larger cohorts of well-characterized patients with NAFLD (as diagnosed by magnetic resonance-proton density fat fraction and magnetic resonance elastography, which are rapidly being recognized as being as good as liver biopsies) [33,34] are certainly needed to better elucidate whether the severity of NAFLD may differentially affect the development of heart block.

Despite these limitations, our study has important strengths, including its large sample size, the ultrasonographic diagnosis of NAFLD, the completeness of the dataset, the ability to adjust for multiple CVD risk factors and potential confounding factors, and the exclusion of patients who took anti-arrhythmic drugs (except beta-blockers), patients with pacemakers or implantable cardioverter defibrillators and those with important comorbidities, such as severe valvular diseases or valve replacement, chronic obstructive pulmonary disease, end-stage renal disease, malignancies or cirrhosis. We believe that the inclusion of patients with such serious comorbidities might have confounded the interpretation of data.

In conclusion, our cross-sectional study showed that ultrasound-diagnosed NAFLD and its severity (using non-invasive markers of advanced NAFLD fibrosis) are strongly and independently associated with an increased risk of prevalent heart block in a large hospital-based sample of adults with T2DM. Future studies are required to better elucidate the biological mechanisms responsible for this association, and to determine whether NAFLD may predict the development and persistence of heart block in people with T2DM.

## Supporting information

S1 Table Clinical and biochemical characteristics of patients with type 2 diabetes stratified by presence or absence of NAFLD on ultrasonography. (DOC)
