# Supplementary material for: Nonalcoholic fatty liver disease is associated with an increased risk of heart block in hospitalized patients with type 2 diabetes mellitus
Source: PLoS One. 2017 Oct 5;12(10):e0185459. doi: 10.1371/journal.pone.0185459 (PMC5628831; doi:10.1371/journal.pone.0185459)
Supplement: S1 Table — (DOC) [file pone.0185459.s001.doc]

**S1 Table.** Clinical and biochemical characteristics of patients with type 2 diabetes stratified by presence or absence of NAFLD on ultrasonography.

| **Characteristics** | **Patients without NAFLD (n=227)** | **Patients with NAFLD (n=524)** | ***P*** |
| --- | --- | --- | --- |
| Age (years) | 67.7 ± 13 | 64.9 ± 12 | <0.005 |
| Men/Women (n) | 110/117 | 295/229 | <0.05 |
| Body weight (kg) | 74.8 ± 19 | 87.1 ± 22 | <0.001 |
| BMI (kg/m^2^) | 27.7 ± 6.5 | 31.6 ± 6.9 | <0.001 |
| Smokers (%), n=219 | 32.3 | 50.6 | <0.05 |
| Diabetes duration (years) | 15 (8-24) | 14 (7-20) | 0.16 |
| Systolic blood pressure (mmHg) | 138 ± 22 | 142 ± 20 | <0.005 |
| Diastolic blood pressure (mmHg) | 77 ± 11 | 81 ± 11 | <0.001 |
| Fasting glucose (mmol/l) | 8.7 (6.8-12.1) | 9.9 (7.1-13.9) | <0.05 |
| Hemoglobin A1c (%) | 9.2 ± 2.6 | 10.0 ± 2.5 | <0.005 |
| Total cholesterol (mmol/l) | 4.20 ± 1.1 | 4.58 ± 1.3 | <0.005 |
| LDL-cholesterol (mmol/l) | 2.41 ± 0.9 | 2.59 ± 1.0 | <0.005 |
| HDL-cholesterol (mmol/l) | 1.13 ± 0.4 | 1.03 ± 0.4 | <0.005 |
| Triglycerides (mmol/l) | 1.36 (1.02-2.03) | 1.74 (1.34-2.56) | <0.005 |
| AST (U/l), n=483 | 18 (14-26) | 20 (16-30) | 0.22 |
| ALT (U/l) | 19 (13-28) | 24 (16-34) | <0.05 |
| GGT (U/l) | 25 (15-48) | 35 (20-60) | <0.05 |
| Creatinine (mmol/l) | 100.2 ± 45 | 103.4 ± 53 | 0.20 |
| Hemoglobin (g/dl) | 12.4 ± 1.8 | 13.2 ± 1.8 | <0.001 |
| Platelets (x 10^9^/l) | 240 ± 80 | 238 ± 69 | 0.60 |
| Hypertension (%) | 74.8 | 83.4 | <0.005 |
| Obesity, BMI ≥30 kg/m^2^ (%) | 26.1 | 52.5 | <0.001 |
| Ischemic heart disease (%) | 23.3 | 20.0 | 0.23 |
| Mild-moderate valvular heart disease (%) | 9.7 | 9.5 | 0.82 |
| Microalbuminuria (%) | 32.8 | 32.0 | 0.79 |
| Macroalbuminuria (%) | 12.9 | 11.0 | 0.34 |
| Diabetic retinopathy (%), any degree | 46.2 | 42.5 | 0.49 |
| Diabetic sensory neuropathy (%), n=731 | 33.8 | 28.7 | 0.18 |
| Peripheral artery disease (%) | 57.7 | 52.0 | 0.09 |
| Insulin users (%) | 70.4 | 71.3 | 0.81 |
| Metformin users (%) | 34.8 | 43.5 | <0.05 |
| Sulfonylurea users (%) | 20.7 | 20.9 | 0.93 |
| Glitazone users (%) | 3.9 | 2.3 | 0.20 |
| DPP-4 inhibitor users (%) | 9.7 | 8.2 | 0.51 |
| GLP-1 analogues users (%) | 0.9 | 1.5 | 0.49 |
| Acarbose users (%) | 3.1 | 2.1 | 0.42 |
| ACE-inhibitor users (%) | 52.4 | 53.0 | 0.87 |
| ARB users (%) | 18.1 | 23.6 | 0.10 |
| Alpha-blocker users (%) | 6.2 | 11.1 | <0.05 |
| Beta-blocker users (%) | 33.0 | 29.4 | 0.34 |
| Dihydropyridine CCB users (%) | 33.9 | 32.4 | 0.70 |
| Diuretic users (%) | 48.0 | 47.5 | 0.91 |
| Anti-platelet drug users (%) | 63.8 | 62.6 | 0.74 |
| Statin users (%) | 55.5 | 63.5 | <0.05 |
| Fibrate users (%) | 2.6 | 4.4 | 0.26 |

Sample size, *n*=751 except where indicated.

Data are expressed as means±SD, medians and interquartile ranges (IQR) or percentages.

Differences between the two groups were tested by the chi-squared test for categorical variables, the unpaired Student’s *t*-test for normally distributed continuous variables or the Mann-Whitney test for non-normally distributed continuous variables.

Abbreviations: ALT, alanine aminotransferase; AST, aspartate aminotransferase; ARB, angiotensin receptor blockers; BMI, body mass index; CCB, calcium channel blocker; DPP-4, dipeptidyl peptidase-4; GGT, gamma-glutamyltransferase; GLP-1, glucagon-like peptide-1.
